# Transforming ‘Bonito del Norte’ Tuna By-Products into Functional Ingredients for Nutritional Enhancement of Cereal-Based Foods

**DOI:** 10.3390/foods12244437

**Published:** 2023-12-11

**Authors:** Adrián Honrado, Paula Ardila, Paula Leciñena, José A. Beltrán, Juan B. Calanche

**Affiliations:** Instituto Agroalimentario de Aragón-IA2-(Universidad de Zaragoza-CITA), Miguel Servet 177, 50013 Zaragoza, Spain; adrihonfri@unizar.es (A.H.); ardilap@unizar.es (P.A.); 778350@unizar.es (P.L.); jbeltran@unizar.es (J.A.B.)

**Keywords:** fish hydrolysate, fishmeal, biscuit, bioactive compounds, fatty acids

## Abstract

The fishing industry produces a significant number of by-products. This study explored two methods of transforming these by-products: fish protein hydrolysate (FPH) and Fishmeal (FM). Physicochemical characterization of these products was conducted and their potential inclusion in biscuits was investigated due to the lack of high biological value protein and polyunsaturated fatty acids of this product. The results identified colour disparities between FPH and FM, with FM displaying lower brightness and a more reddish hue. In FPH, there was also a noticeable decrease in polyunsaturated fatty acids, probably associated with the temperature reached in spray-drying. While the incorporation of these by-products in biscuits was feasible, there were challenges, particularly the fishy taste and rancid odour, which were more pronounced in FM biscuits due to the higher fat content. This correlated with the oxidation indexes, such as TBARS and acidity index. Nonetheless, FPH biscuit attributes like typical colour or flavour received positive feedback, attributed to the Maillard reaction. Scanning electron microscopy revealed microstructural differences, which correlated with the results of hardness and fracturability, probably due to the higher fat content in FM. This study revealed the possibility of nutritionally enriching cookies with ingredients derived from fish by-products. However, it would be necessary to go a step further and study alternatives that allow better preservation of saturated fatty acids.

## 1. Introduction

Tuna, a highly prized and globally consumed fish, has a significant economic and nutritional value. Global catches of tuna and tuna-like species have continued growing in recent years, although catches declined to 7.8 million tonnes in 2020 due to COVID-19 [1]. In Spain, 33,530 t were caught in 2021, 12.9% more in comparison to 2020 [2]. Tuna is also valued for its optimal nutritional profile. Every 100 g of raw tuna is estimated to contain around 119 kcal, 22 g of high biological value protein and approximately 3.3 g of fat [3]. In this sense, tuna contains a large amount of Ω3 polyunsaturated fatty acids, mainly EPA (eicosapentaenoic acid) and DHA (docosahexaenoic acid) [4,5]. Among other benefits, they have been associated with a reduced risk of cardiovascular diseases as they could decrease triglyceride and plasma triglyceride concentrations, platelet aggregation, and blood pressure [6,7]. Tuna is also an excellent source of key nutrients such as vitamin D, vitamin B12, selenium and phosphorus [3].

In the case of Spain, the fish canning industry is a sector with a long tradition and an interesting market segment. In particular, ‘Bonito del Norte’ (*Thunnus alalunga*) has a crucial role in the economic and cultural panorama of the country. The canning tradition, rooted in North Spain regions, has passed down from generation to generation, combining artisanal techniques with technological advances to guarantee maximum product quality. Based on official data from FAO [2], the industry not only bolsters the Spanish economy by generating thousands of direct and indirect employment opportunities but also plays an important role in the nation’s export portfolio due to the high demand for white tuna products in global markets. Furthermore, the industry’s significance is underscored by its commitment to sustainability. Given the substantial volume of trimmings and waste produced, stringent regulations have been instituted, covering both bonito fishing and processing. These measures aim to safeguard marine resources and uphold the enduring legacy of this cherished tradition.

As noted above, tuna processing results in substantial by-products, which include the head, bones, skin, viscera, and other parts that are often discarded. These by-products represent 30–50% of the total fish weight, and in the case of tuna, this percentage rises up to 70% [6,8]. Specifically, two decades ago, the fish filleting sector generated around 3.17 million tonnes of waste [9], and today, it reaches 22 million tonnes [2]. Due to this increase, sustainability and efficient resource utilisation have become critical concerns in the seafood industry, and finding innovative ways to use these by-products is gaining attention. Repurposing tuna by-products can be useful for the development of valuable secondary products such as fishmeal, fish oil, pet food and, in recent years, functional ingredients for humans [10]. This would promote the circular economy and some of the sustainable development goals: no hunger, good health and welfare, responsible consumption and production, and underwater life [11,12]. Furthermore, they would provide interesting nutrients to those who consume such products.

In this sense, the production of fishmeal (FM) or fish protein hydrolysates (FPH) could be interesting alternatives for the utilisation of these by-products in human nutrition. In the first case, the meat is minced, boiled, partially defatted, and then dried, obtaining a product with high protein content, between 75% and 95% [13]. In the second case, by-products are subjected to an enzymatic treatment that breaks down the proteins into smaller peptides. Subsequently, the enzyme is inactivated by heat, and the fat content is standardised and spray-dried [14]. This second alternative can also produce peptides with positive effects on skin and bone health, blood lipid profile, or weight control, among others [15,16]. Further, both alternatives contain a large amount of Ω3 polyunsaturated fatty acids, mainly EPA and DHA.

These products, with new functional ingredients obtained from by-products, could perfectly enrich existing foodstuffs that are poor in high biological value protein and polyunsaturated fatty acids. An example of such products is biscuits: foodstuffs made from a mixture of flour, edible fats, and water, with or without added sugars and other foodstuffs (additives, flavourings, seasonings, spices, etc.), subjected to a kneading process and subsequent heat treatment, resulting in varied presentation products characterised by its low water content [17].

The origin of biscuits goes back 10,000 years. At this point, our nomadic ancestors discovered that cereal dough that was heated to a consistency like unleavened bread could be easily transported, especially during the long journeys of that era [18]. With the industrial revolution, the first sea biscuit factories appeared, especially in the United Kingdom and France. However, the demand for this type of biscuit declined around 1830 due to the introduction of steam in shipping. This reduced journey times considerably, and it was no longer so important to have large quantities of biscuits on board. British manufacturers had to find new customers, so they added cocoa, sugar, butter, and milk to the dough to make the product more attractive to consumers [19]. Nowadays, biscuits are mostly consumed as a food for pleasure rather than an indispensable product in human nutrition. Moreover, there is also a certain predilection for this product in the case of children.

Biscuits’ nutritional quality depends on the nature and quantity of the ingredients used. Biscuits are generally high in carbohydrates, fat (predominantly saturated), and calories but low in protein, fibre, vitamins, and minerals [20]. Furthermore, protein content in wheat flour is 7–14%, with a lack of some amino acids such as lysine [21]. In this sense, some research shows a relation between biscuit intake and overweight status. It has been demonstrated that each extra megajoule of energy from biscuits, cakes, and confectionaries increased the odds of being overweight by 24% in children aged 7–18 years old [22]. However, it is true that obesity must be tackled from a multidisciplinary approach that considers physical activity as well as eating habits.

The development of new, healthier foods or the incorporation of ingredients with some beneficial effects on health could help to mitigate this situation. Several studies have developed new biscuit formulations through the incorporation of different products such as buckwheat, grape, pumpkin, or copra, among others, in an attempt to make them a healthier product [23]. However, biscuits enriched with fish-origin compounds are less common and could be an excellent option to improve their nutritional profile. Therefore, the primary aim of this study was to produce and characterize FM and FPH derived from ‘Bonito del Norte’ tuna (*Thunnus alalunga*) by-products sourced from Spanish Canning Industries. Furthermore, we aimed to assess the viability of incorporating these products as functional ingredients in biscuit manufacturing, with the goal of enhancing nutritional value through enrichment with unsaturated fatty acids while simultaneously ensuring satisfactory sensory quality.

## 2. Materials and Methodology

### 2.1. Obtaining Functional Ingredients (FPH and FM)

**Figure 1:**
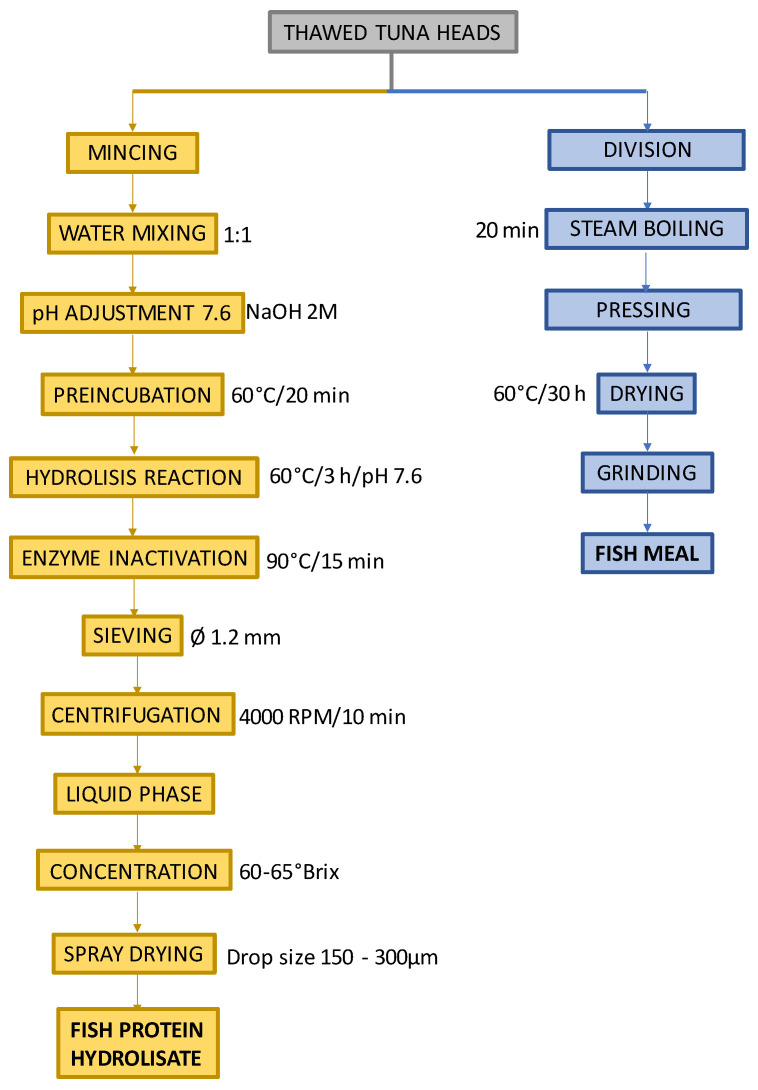
Procedure for the obtention of FPH (fish protein hydrolysate) and FM (fishmeal).

In the production process of both ingredients, *Thunnus alalunga* heads supplied by BARNA SA were used. The FPH and FM procedure followed is shown in Figure 1.

The thawed fish heads were minced in a cutter (Sammic mod. SK-3, Azkoitia, Spain), and then the paste obtained was mixed with distilled water in equal parts in a 2 L Erlenmeyer flask. It was then placed in a water bath at 60 °C, and using a pH meter (Crison mod. pHmeter basiC 20, Alella, Spain), the pH was adjusted at 7.6 with NaOH 2M (Panreac Química SLU, Castellar del Vallés, Spain). The enzyme was added at 0.5% (*w*/*w*). The decrease in pH produced by the hydrolysis was adjusted with NaOH 2M, maintaining the pH at 7.6. The hydrolysis procedure ended when there was no pH decrease anymore (3 h). The enzyme was then inactivated by heat (90 °C and 15 min), and the hydrolysate was cooled to room temperature. The mixture was sieved through a sieve (Ø 1.2 mm), and the liquid part was centrifuged at 4000 rpm for 10 min to obtain 3 phases: sludge (lowest phase), aqueous protein phase (middle phase), and lipid phase (upper). The lipid phase was removed using a Pasteur pipette, and the liquid phase, which also contained part of the phospholipidic fraction, was atomised with a droplet size between 150 and 300 µm and a temperature of 220 °C. To produce the FM, the thawed heads were cut into smaller pieces, steam-boiled, pressed, and then dried in an oven at 60 °C with forced convection for 30 h (Verinox mod. Junior 1100, Altopiano della Vigolana, Italy). The last step was to grind them until it became a powder (Moulinex mod. Moulinette A320R1, Alençon, France).

### 2.2. FPH and FM Characterization

#### 2.2.1. Colour

The colour of the FPH and FM was determined using a colorimeter (Minolta mod. CM-2002, Tokyo, Japan). Colour evaluation was expressed in CIEL*a*b* coordinates, where L* refers to lightness, from 0 (black) to 100 (white); a* represents the variation from green (−128 to 0) to red (0 to 127); and b* represents the variation from blue (−128 to 0) to yellow (0 to 127) [13,24]. The colour difference was calculated according to a study by Ainsa et al. [12] using the total colour difference (ΔE) between the FPH and the FM and a control. Wheat flour was considered as the control.

#### 2.2.2. Fat Content, Lipid Profile, and Lipid Oxidation

As one of the main advantages of incorporating fish by-products in biscuits is the increase in the Ω3 fatty acid content, mainly ALA, EPA, and DHA, it was decided to carry out a comparison of the content and profile of the raw material, FPH, and FM.

First, the lipid content of raw material, FPH, and FM were determined following methodology AOCS 920.39C [25]. Fatty acid profiles of raw material, FPH, and FM were determined according to Bligh and Dyer [26]. Firstly, each sample was homogenised with an Ultraturrax device (IKA mod. T-25 basic, Staufen, Germany) using different solvents such as chloroform, methanol, potassium chloride (CarloErba, Sabadell, Spain), and water. This mixture was centrifuged for 10 min at 4000 RPM (Hettich mod. Universal 320 R, Tuttlingen, Germany), and the fat was extracted from the lower phase. Then, after incorporating BHT (Sigma Aldrich, Saint Louis, MO, USA, EEUU) as an antioxidant substance, solvents were evaporated with nitrogen gas. Afterwards, methylation was performed using 0.03 g of this previous fat. This fat was mixed with an intern pattern, C23:0 (TCI, Tokyo, Japan), which did not cause interferences in the matrix. Then, 2 mL of hexane and 1 mL of potassium hydroxide (Merck, Darmstadt, Germany) saturated solution in methanol were added. The upper phase was extracted. A gas chromatograph (Hewlett-Packard mod. 6890 II, Palo Alto, Santa Clara, CA, USA, EEUU) was employed with an SP-2380 (100 m × 0.25 mm × 0.20 µm) column. The temperature program was 140–165 °C at 3 °C/min for 10 min and 165–220 °C at 5 °C/min for 50 min. Fatty acid content was quantified as total area (%) of identified fatty acids.

For lipid oxidation, the TBARS (thiobarbituric acid reactive substances) were determined in raw material, FM, and FPH following the methodology cd 19-90 [25]. This method is based on the sample distillation in acid medium, followed by a reaction of the distillate with thiobarbituric acid. Absorbances were measured at 532 nm, and concentration was determined by interpolation of the samples on a calibration curve made with thiobarbituric acid and increasing concentrations of tetramethoxypropane (Merck, Darmstadt, Germany). The results were expressed as mg malonaldehyde per kg sample.

### 2.3. Biscuits Manufacturing Process

The ingredients used for biscuit manufacturing are described in Table 1. The ingredients were mixed, first with a spiral beater (Silvercrest, Neckarsulm, Germany) and manually after that. The dough was rolled out with a rolling pin on a smooth surface covered with flour, and the moulds were placed on top of it to form circular biscuits. The biscuits were placed in the oven (Salva mod. LT-4+H 00, Lezo, Spain) at 170 °C for 14 min. FM and FPH substitution percentages were carried out on the basis of tests performed previously.

Once the biscuits were baked, they were left to cool to room temperature and packaged in plastic trays covered with a waterproof film. Three batches following the same procedure were produced on subsequent days. Each batch and treatment contained 15 biscuits. The diameter of the biscuits was 6 cm, the thickness was 0.4 cm, and the weight was 7.1 g.

### 2.4. Biscuits Characterization

#### 2.4.1. Physicochemical Characterization: Colour, Lipid Oxidation, Moisture, and a_w_

The procedure followed was the same as that carried out with the FPH and FM. In this case, the colour was measured on the upper part of the biscuits. For the ΔE calculation, the control biscuit was used as a reference.

Acidity index assay was conducted on biscuits following ISO 660:2020 standard [27], expressing the results in percentage of oleic acid. Lipid oxidation was determined following the methodology cd 19–90 [25].

The moisture of the biscuits was determined using a thermogravimetric balance (KERN & SOHN GmbH, Balingen-Frommern, Germany), introducing between 1 and 1.5 g of previously crushed sample in a mortar. Water activity was determined instrumentally (Decagon Devices mod. Aqualab CX-1, Pullman, WA, USA).

#### 2.4.2. Textural Characterization

The texture of the biscuits was assessed instrumentally using a texturometer (Stable Micro Systems mod. TA XT2i, Godalming, UK) with a stainless steel cylindrical P2 probe to perform a penetration test. The biscuits were placed on a platform, and the 2 mm cylindrical probe was fitted to the instrument. The texturometer was set to a return cycle at start-up, with a speed of 10 mm/s and 5 mm distance between the 50 kg load cell and the biscuit [13]. The parameters of strength (g) and fracturability (mm) were obtained. The maximum force to perform a deformation on the biscuits with the teeth when chewing was determined. On the other hand, fracturability was used to measure the force needed to break the biscuit [28]. This corresponded to the distance (mm) to the maximum force (g).

#### 2.4.3. Sensory Characterization

The biscuits’ sensory analysis was carried out in the tasting room of the Pilot Plant of the Faculty of Veterinary Medicine, complying with ISO 8589:2007 [29]. A panel of 10 trained sensory evaluators (ISO 8586:2023) was employed [30]. These sensory evaluators had previously demonstrated sensory sensitivity in preliminary tests, received considerable training, and were able to make consistent and repeatable assessments of various commercial biscuit samples. This also allowed the assessors to acquaint themselves with the attribute terms and the scoring system. In this sensory analysis, a quantitative descriptive analysis (QDA) was used after defining a specific sensory profile. The selected attributes were chosen by mutual agreement between the sensory panel director and the assessors based on commercial samples and the existing literature [20,31]. Then, the ‘Comparison with a Reference’ method was used [32]. A total of 14 attributes were assessed: aspect, porosity, homogeneity, typical biscuit colour, typical biscuit flavour, oil smell, rancid odour, fracturability, hardness, salty flavour, fish flavour, rancidity flavour, and off-flavour.

In this test, judges evaluated the samples against a reference (ISO 13299:2016) [33], which in this case corresponded to the control biscuit. Three sessions were carried out on three consecutive days, with a time lapse between manufacture and sensory analysis of less than four days. A non-structured but end-anchored scale was used for quantification. For this, the reference sample (R) was placed in the middle of the scale, presenting a ± 5 cm distance to the ends from the reference. In the execution of the test, the samples were always presented in pairs to each trained sensory evaluator, following a balanced and equilibrated design. They were asked to assess by contrast (direct comparison) the magnitude of the change of each sensory attribute (more or less than the reference).

#### 2.4.4. Biscuit Overview (Global Analysis)

To obtain a visual interpretation that allowed us to observe the relationships between the samples and the variables, as well as the relationship between sensory and physicochemical variables (moisture, a_w_, colour, texture, and oxidation parameters), a PCA (principal component analysis) was carried out. For this, the descriptors that were significant in the sensory analysis and all the physicochemical variables were used.

#### 2.4.5. Microstructure Characterization

The microstructural behaviour was analysed in both control and enriched biscuits using a Scanning Electron Microscope (JEOL mod. JSM 6360-LV, Tokyo, Japan). A cross-section of the biscuit was Au/Pd-coated and then observed at 500× magnification and 15 kV [34]. This allowed us to visualise the internal structure of the biscuit and to find the explanation for certain behaviours observed in the texture profile and sensory analysis.

### 2.5. Statistical Analysis

The results obtained were analysed with the XLSTAT 2016 Statistical Software [35]. Analyses performed included the calculation of maxima, minima, quartiles, medians, means, variances, box plots, scatterplots, and normality tests.

For the physicochemical parameters, an ANOVA was carried out. This model included treatments as fixed variables and the three replicates as a random effect. The interaction between them was also studied. Approximate F-ratio tests for each fixed effect were conducted, and the critical value for a statistical effect was taken at *p* < 0.05. A pairwise comparison between means was carried out using Dunnett’s multiple comparisons test.

For the sensory data, a panel analysis, which included the judge, session, product effects, and their interactions as variables in the model, was performed. This model also considered the random session and product effect and was useful for detecting and removing panellists far from the consensus. Then, an analysis of variance was carried out on the sensory data, using Fisher’s test (LSD) as an a posteriori test. All the attributes of the control biscuits were scored as 0 as they were the reference sample. This model included treatments as a fixed variable, the replicate as a random effect, and the interaction between them.

To obtain a simultaneous representation that allowed a better visualisation of which biscuits were the most and least like the control one, a ranking table was made. For this purpose, only the descriptors in which significant differences were found were used. For each of them, the biscuits were ordered according to the mean value for each significant descriptor: 5 for the control biscuit and 1 for the biscuit least like the control. From this table, a similarity/similarity matrix was constructed based on the Euclidean distance, applying the Ward method as an agglomeration technique. The proximity matrix obtained was used to perform multidimensional scaling (MDS) with the following parameters: absolute model, Kruskal stress, 2 dimensions, random initial configuration, 5 repetitions, convergence of 0.00001, and 500 iterations. This method is often used in psychometrics (perception analysis) and marketing (distances between products obtained from consumer ratings). However, it is gaining more and more ground in the field of sensometrics applied to food [36].

## 3. Results

### 3.1. FPH and FM Characterization

#### 3.1.1. Colour

**Figure 2:**
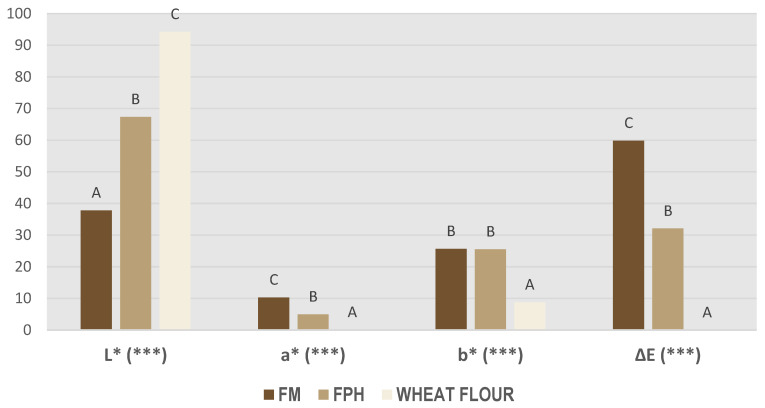
CIEL*a*b* and ΔE parameters for hydrolysates (FPH) and fishmeal (FM). Different letters show significant differences among samples. *** *p* < 0.001.

Figure 2 shows CIEL*a*b* and ΔE parameters for FPH and FM. Significant differences were not observed among the replicates, and the interaction between fixed effects was not significant. In relation to the L* coordinate, it can be observed that wheat flour was the one with the highest value, because of its whiter colour. FM was the one with the lowest L*, which could be related to fat and haemoglobin presence and mainly to the presence of dark-coloured parts that are eliminated in the hydrolysate processing by filtration and centrifugation processes but not in fishmeal production.

In a* coordinate, the highest red indices were found in the FM, mainly due to the presence of haemoglobin and reddish tones acquired by the drying process and the presence of dark-coloured parts [37]. The b* coordinate showed no significant differences between FM and FPH. Finally, ΔE was twice as high for FM as for FPH. Therefore, the use of FM instead of FPH would lead to a greater colour variation in the product in which it is incorporated.

#### 3.1.2. Fat Content, Lipid Profile, and Lipid Oxidation

The determination of the fat content showed that the FPH contained a lower amount because of the defatting process after hydrolysis. Therefore, the tuna FPH obtained an average value of 0.85%, mainly from phospholipids. On the other hand, the FM showed a higher fat content of 9.96% (*p* < 0.05). Significant differences were not observed among the replicates, and the interaction between fixed effects was not significant.

On the other hand, Table 2 shows the fatty acid profile of raw material (tuna heads), FM, and FPH. 

In relation to saturated fatty acids, a significant increase in the sum of saturated fatty acids was perceived (*p* < 0.05), with FM having the highest value: 3% more than the raw material. However, lignoceric acid was the one that obtained the highest increase when comparing the raw material with the FPH. For monounsaturated fatty acids, a significant increase in the total sum was observed, especially when comparing the raw material with the FPH. The explanation lies in two fatty acids: oleic, which decreases by 84.54% and vaccenic, which is 10.99 times higher. This is an example of positional isomerism, as vaccenic is the positional isomer of oleic acid [38]. Furthermore, the research of Routray et al. (2018) concluded that temperature was observed as a noteworthy factor with significant linear and/or quadratic effects in the formation of this fatty acid [39]. 

In relation to polyunsaturated fatty acids, there is a notable decrease when comparing the raw material with FM and FPH: 4.97% and 18.25%, respectively. This affects FPH especially because of the heat treatment applied when spray-drying and mainly involves EPA and DHA, which decreased 38.87% in the case of the first and 61.3% for the second when comparing the raw material with FPH [40]. These results are in accordance with TBARS results and confirm the oxidation process that takes place. Similar behaviour was found in the study of Gómez-Guillén et al. (2022), in which, despite the use of antioxidants, the decrease in these fatty acids was more pronounced when spray-drying was used in comparison to traditional forced convection drying [41].

Furthermore, a target of Ω-6/Ω-3 fatty acid ratio of 1:1 to 2:1 appears to be consistent with studies on evolutionary aspects of diet, neurodevelopment, and genetics. A lower Ω-6/Ω-3 fatty acids ratio is more desirable in reducing the risk of many of the chronic diseases (coronary heart disease and possibly other chronic diseases) of high prevalence in Western societies, as well as in developing countries [42]. In this case, tuna FPH was the closest to the recommended value. On the other hand, according to several studies, the p/s ratio should be as close to 1 as possible [43]. Both the FPH and the FM obtained values very close to 1. Significant differences were not observed among the replicates, and the interaction between fixed effects was not significant.

The TBARS value for the raw material was 1.16 mg MDA/kg. The highest value was found in the tuna FM (3.46 mg MDA/kg) followed by the tuna FPH (1.64 mg MDA/kg), all of them significantly different (*p* < 0.05). Significant differences were not observed among the replicates, and the interaction between fixed effects was not significant. This was an increase of 2.98 and 1.41 times over the raw material, respectively. Indeed, the unsaturated character of the fat, which has been observed in the fatty acid profile, as well as the haemoglobin iron content and the application of heat, could be responsible for these increases compared to the raw material [44,45,46]. However, it should be considered that the fat content of FM is higher, so the higher TBARS index in FM does not mean that the FM obtention process produces higher oxidation. In fact, if TBARS values are expressed exclusively in relation to fat, a significantly higher TBARS index is observed in FPH.

### 3.2. Biscuits Characterization

#### 3.2.1. Physicochemical Characterization: Colour, Lipid Oxidation, Moisture, and a_w_

Table 3 shows the physicochemical characterization of biscuits. Relating to colour, the incorporation of fish in the different treatments resulted in a decrease in lightness, especially with the treatments that incorporated FPH (*p* < 0.05). However, the a* coordinate increased, which was again more noticeable in those treatments containing FPH, although in the colour analysis of the FPH and the FM, the second obtained a higher a* coordinate. Finally, the b* coordinate behaved similarly to a*. In general, the biscuits differed from the control by having a lower level of white and a higher red-yellow colour. Similar behaviour was observed in the biscuits made by Mohamed et al. with carp and shark FPH [47]. This fact could have two causes. On the one hand, the colour of the FM and FPH that are used could influence the results. On the other hand, caramelisation and Maillard reaction [48]. Maillard reaction is related to the high protein content, especially in FPH, whose protein reacts with reducing sugars, resulting in the above-mentioned variations [49]. Variations in coordinates caused by the addition of FM or FPH were also studied by ΔE. This value was higher in the treatments that included FPH, given its higher protein content and its characteristic colour. Significant differences were not observed among the replicates, and the interaction between fixed effects was not significant.

In relation to the biscuit’s lipid oxidation indexes, higher acidity could be observed in the treatments containing FPH compared to those containing FM, which indicates some degree of hydrolysis of the fatty acids. This could be a consequence of the enzymatic and thermal treatment carried out to obtain the product. TBARS values were lower in the treatments that included FPH as its fat content was lower (Table 3). However, these values might not be detectable to the senses, as some researchers, like Connell [50], suggest threshold levels ranging between 1 and 2 mg MDA/kg. No significant differences were observed for both the acidity index and TBARS among the replicates, and there was no significant interaction between the fixed effects.

Finally, regarding moisture content, no significant differences were found with respect to the control. However, there is a trend for biscuits containing FPH to have higher moisture values. These could be an effect of fish peptides. They have water-soluble hydrophilic peptides, so free water can be trapped in biscuit dough, showing higher moisture content [51]. The a_w_ did not show significant differences with respect to the control biscuit. Significant differences were not observed among the replicates, and the interaction between fixed effects was not significant.

#### 3.2.2. Textural Characterization

As can be seen, both fracturability and hardness were decreased when substituting with FM or FPH (Table 2). The decrease was greater as the percentage of substitution increased. However, no significant differences were established (*p* > 0.05), except for FPH-5 (*p* < 0.01). These decreases could be related to lower gluten concentration and interaction with wheat proteins or even to the extra fat content from FPH and FM that weakens the protein structure [52]. This resulted in a weaker and less elastic dough. Significant differences were not observed among the replicates, and the interaction between fixed effects was not significant.

#### 3.2.3. Sensory Characterization

**Figure 3:**
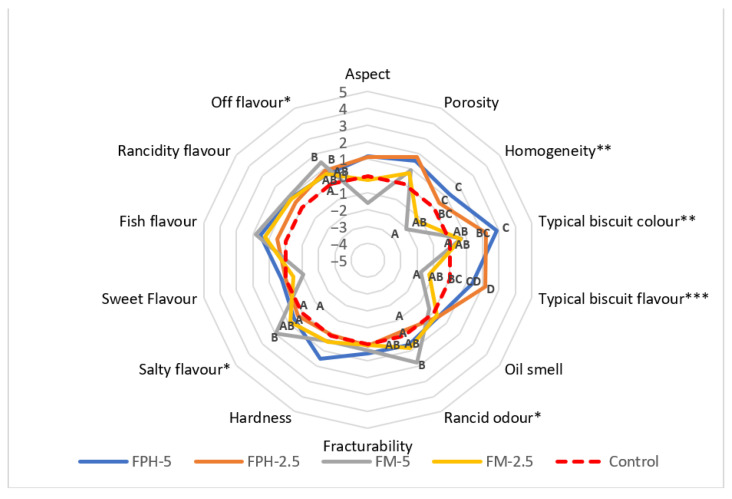
General sensory profiles for enriched biscuits based on quantitative descriptive analysis. FPH: fish protein hydrolysate; FM: fishmeal. The numbers 2.5 and 5 refer to the percentage of wheat flour substitution. * *p* < 0.05; ** *p* < 0.01; *** *p* < 0.001.

The sensory analysis revealed distinct variations in 6 of the 14 evaluated attributes: homogeneity, typical biscuit colour, inherent biscuit flavour, rancid odour, salty taste, and off-flavour (as depicted in Figure 3).

Biscuits incorporating fishmeal (FM) exhibited pronounced disparities (*p* < 0.01) in their homogeneity, contrasting with control biscuits and those containing FPH, which displayed no significant differences. This phenomenon likely arises from FPH’s particle size, closely resembling that of wheat flour. Notably, biscuits containing FPH, particularly the FPH-5 treatment, exhibited a more distinct characteristic colouration compared to the others. This phenomenon can be attributed to FPH’s elevated protein content, which enhances the formation of melanoidins through the Maillard reaction, as elucidated by Wang et al. [53]. Similar outcomes were reported by Cai et al. [54] in a related study involving shrimp waste hydrolysate, where substantial alterations in Maillard reaction products (MRPs) occurred, including the accumulation of intermediate or browning products, yielding a visually appealing reddish-brown hue closely associated with a robust and enticing flavour and colour profile.

Considering the inherent biscuit flavour, those infused with FPH exhibited a higher intensity, with the FPH-2.5 variant standing out prominently. This sensory enhancement can be attributed to the Maillard reaction and the consequent generation of flavour compounds that harmonize with the traditional biscuit taste, as documented by Martins et al. [48]. Conversely, biscuits containing FM displayed a reduced biscuit flavour. This phenomenon may be attributed to a twofold effect: firstly, the flavour compounds generated by the Maillard reaction in FM-based biscuits are possibly masked by any fishy undertones, and secondly, the fish flavour may be altered due to the hydrolysis reaction. Importantly, biscuits with a muted characteristic flavour exhibited a pronounced off-flavour. The above may be linked to an elevated fishiness in FM biscuits or heightened oxidation levels. This observation aligns with the rancid odour attribute, where the FM-5 biscuit markedly differed from its counterparts. This would be related to the high fat content of FM [55]. The final significant attribute was the salty taste, where the FM-5 treatment distinctly deviated from both the control and FPH-based biscuits. The sensory analysis highlights the merits of integrating FPH into biscuit enrichment. It amplifies the desirable characteristics while ensuring that any negative traits remain comparable to the control. Interestingly, judges could not discern differences in attributes like hardness, which was statistically significant in the instrumental texture assessment. Likewise, in terms of porosity, while the SEM revealed changes upon adding FPH or FM, the judges could not detect any differences.

**Figure 4:**
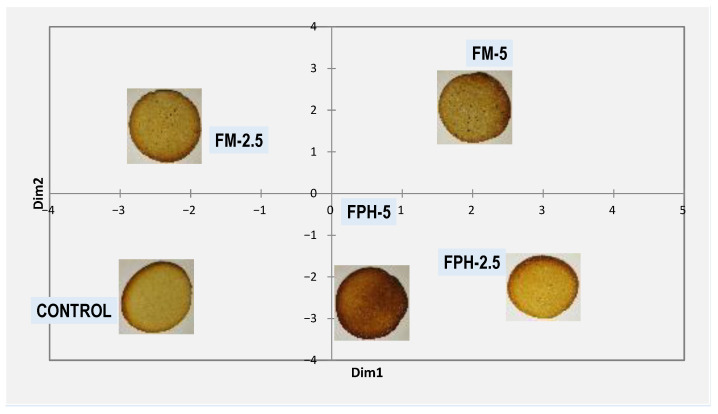
Multidimensional scaling analysis of the different biscuits considering only significant descriptors (*p* < 0.05). FPH: fish protein hydrolysate; FM: fishmeal. The numbers 2.5 and 5 refer to the percentage of wheat flour substitution.

Looking at the multidimensional scaling graph (Figure 4) estimated from the trained sensory assessors based on their assessments, considering only the attributes that were significant, the most similar biscuit to the control was FM-2.5. In the first dimension (D1), the control biscuit was located opposite to both FPH (2.5% and 5%) and FM-5. In this sense, FPH-2.5 and FPH-5 stood out for their homogeneity, typical colour, and absence of rancid odour, while FM-5 was probably penalised for its low homogeneity, typical odour intensity, and high rancid odour. Nonetheless, in the second dimension (D2), it can be seen how enzymatic hydrolysis could provide ingredients which, once inside the enriched biscuits, were able to keep the similar organoleptic properties that those that reference biscuits made with typical ingredients; this closeness would be due to its overall appearance and typical aroma. These findings confirmed the results obtained by other authors who indicated that the conversion of low-value fish-derived materials, such as fish powder, into more valuable products, such as flavour precursors and subsequent flavour compounds, might be a commercially viable proposition for the fish industry [56].

#### 3.2.4. Biscuit Overview

**Figure 5:**
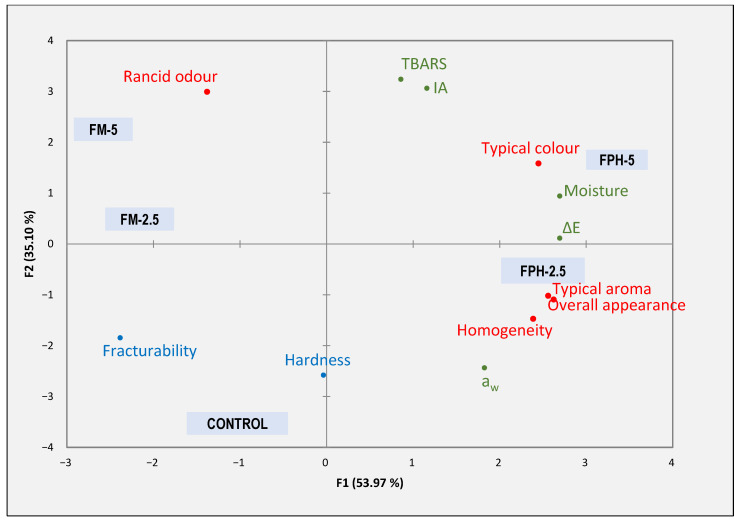
PCA (principal component analysis) of biscuits containing FPH and FM. FPH: fish protein hydrolysate; FM: fishmeal. The numbers 2.5 and 5 refer to the percentage of wheat flour substitution. Red, blue and green colours refer to sensory, instrumental texture and physicochemical parameters respectively.

Figure 5, employing principal component analysis (PCA), provides a comprehensive exploration of the relationships between various enriched biscuits in terms of sensory attributes and physicochemical parameters. The analysis captured a significant 89.07% of the data’s total variability. The first principal component, representing 53.97% of the variance, differentiated FM biscuits and control biscuits from FPH biscuits. Notably, FM biscuits correlated strongly with oxidative traits such as rancid odour and oxidative indices like TBARS and IA. In contrast, the control biscuit, while predominantly characterized by its hardness and fracturability, shared certain sensory features, including aroma, homogeneity, and overall appearance, with the FPH-2.5 biscuit. The second principal component, accounting for 35.10% of the variability, predominantly associated physicochemical variables with FM biscuits, particularly those related to oxidation. Intriguingly, the FPH-5 variant, although leaning towards oxidation, stood out for its colour attributes and ΔE, signifying a notable shift in colour, especially when compared to the control. Collectively, this analysis sheds light on the clear distinctions between biscuit types, guiding optimal enrichment strategies to balance sensory appeal with nutritional benefits.

#### 3.2.5. Microstructure Characterization

**Figure 6:**
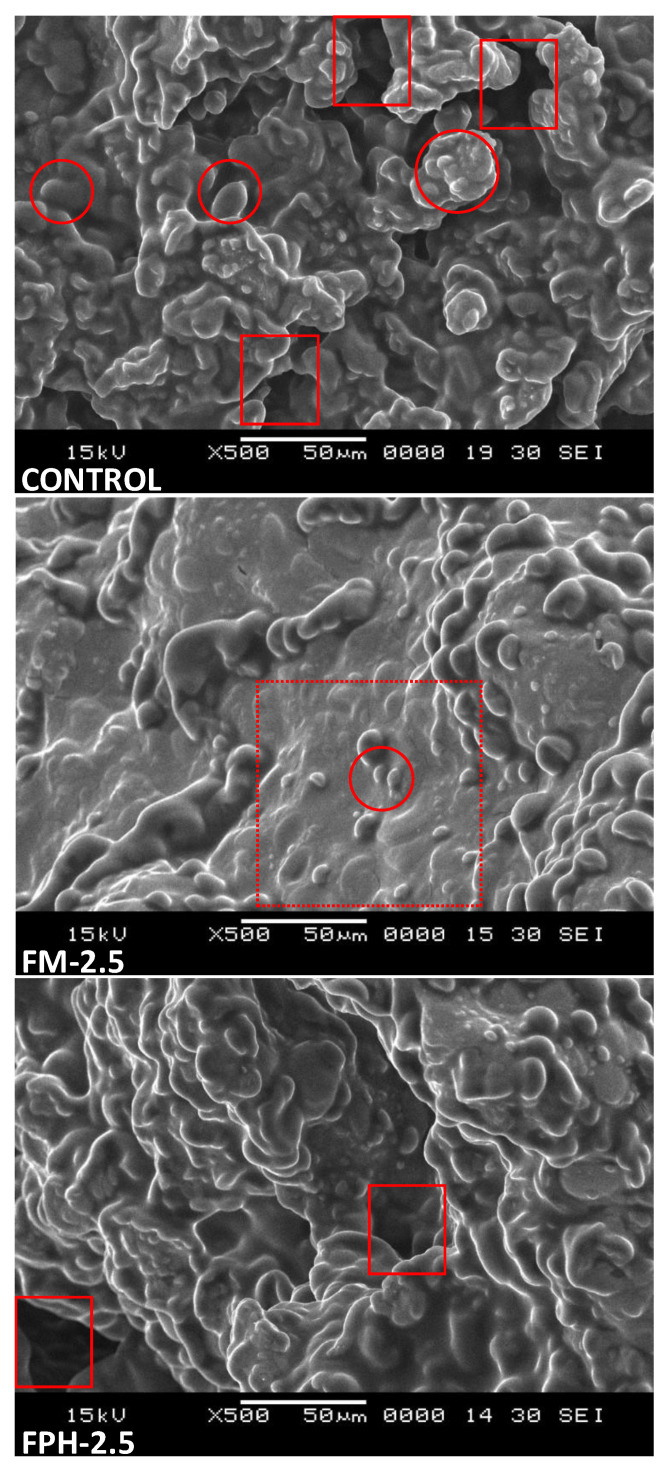
SEM images of control biscuit FPH-2.5 and FM-2.5. FPH: fish protein hydrolysate; FM: fishmeal. The numbers 2.5 and 5 refer to substitution percentage. Circles show partially gelatinized starch granules. Rectangles show hollow areas. The rectangles with dashed lines show areas where the protein/fat matrix is clearly observed.

Figure 6 shows the scanning electron microscopy (SEM) of both the control biscuits and FM-2.5 and FPH-2.5 biscuits, as according to the PCA, they were considered the most similar ones to the control. This study made it possible to study the internal changes in the biscuit structure (particle size or uniformity, among others) that may affect the sensory characteristics. In the control biscuit, the predominance of spherically shaped structures can be observed (marked with circles in Figure 6). These shapes are characteristic of partially gelatinized starch granules since, due to the existence of sugars in the dough and the small amount of water, gelatinization is not completed, and a linear structure is not created [57]. In addition, greater porosity is also observed, with dark zones indicating a greater depth, which translates into air-filled zones, increasing the porosity of the biscuit (marked with rectangles in Figure 6). On the other hand, the biscuit with 2.5 tuna FPH is the one that perhaps obtained an aspect more similar to the control, with certain darker zones due to a greater porosity. However, it can be observed how the starch granules are partially embedded in a continuous phase that would be formed mainly by sugars, fat, and peptides [58].

The biscuit enriched with FM displayed the most pronounced differences compared to the control. These FM biscuits were defined by a continuous phase composed of protein and fat, from which starch granules subtly protruded, nearly appearing ‘submerged’ (highlighted within dashed rectangles in Figure 6). This distinct phase is attributed to FM’s higher fat content relative to biscuits infused with FPH and the presence of proteins over peptides [59]. Yet, the absence of air pockets does not necessarily imply increased hardness. The fat occupying these voids could disrupt interactions between starch and protein particles, potentially resulting in reduced hardness, a finding supported by both sensory and instrumental texture analyses [60]. In this context, the texture profile analysis (TPA) concurred with the scanning electron microscopy (SEM) observations, noting variations in biscuit fracturability tied to their rheological properties, as evident in the images. However, the sensory analysis revealed so few differences in fracturability that the sensory assessors could not discern among them.

## 4. Conclusions

It can be concluded that the inclusion of fish protein hydrolysate (FPH) and Tuna Fishmeal (FM) in the formulation of cookies was feasible. Additionally, this research has yielded other conclusions. Regarding the production of FPH and FM, it was determined that the temperature reached during the spray-drying process used in FPH manufacturing negatively affects the essential fatty acids ALA, EPA, and DHA. This also resulted in a higher TBARS index, considering the lipid content of both FPH and FM. Conversely, obtaining FM without removing non-protein fractions led to darker tones. Concerning the cookies, the importance of the Maillard reaction in this product was concluded. The addition of FM and FPH favoured a higher degree of toasting under the same baking conditions, especially in cookies with a higher protein content, such as those with FPH. Cookies with FPH exhibited higher moisture content, related to the presence of hydrophilic peptides, which could also affect fracturability and texture properties because, as could be seen in SEM images, structural changes were produced when adding FPH or FM. Sensorially, it is noteworthy that cookies with FPH were associated with a stronger flavour and typical colours, perhaps a consequence of compounds generated in the Maillard reaction. Additionally, sensory analysis left no doubt about the susceptibility of fatty acids to oxidation. In summary, the preliminary products developed in this article pave the way for a new line of research that could result in a stable product with greater nutritional richness where good consumer acceptability must be achieved. Undoubtedly, looking ahead, aspects to consider for future research in this field include the use of antioxidants, modification of FPH obtaining techniques with methods requiring lower temperatures (e.g., vacuum evaporation), and the use of a consumer sensory panel to assess acceptance levels.

## Figures and Tables

**Table 1:** Ingredients (%) used for development of enriched biscuits.

	CONTROL	FPH-2.5	FPH-5	FM-2.5	FM-5
Wheat flour	51.8	49.3	46.8	49.3	46.8
Tuna FPH	0.0	2.5	5.0	0.0	0.0
Tuna FM	0.0	0.0	0.0	2.5	5.0
Sugar	15.6				
Sunflower oil	17.3				
Milk	13.8				
Salt	0.5				
Baking powder	0.5				
Vanilla flavouring	0.5				

FPH: fish protein hydrolysate; FM: fishmeal. The numbers 2.5 and 5 refer to percentage of flour substitution.

**Table 2:** Fatty acid profile of raw material, tuna FPH (fish protein hydrolysate), and tuna FM (fishmeal).

Fatty Acid (g FAME/ 100 g Sample)	Raw Material	Tuna FM	Tuna FPH
C14 ***	3.117 ^c^	2.375 ^a^	2.794 ^b^
C15 **	0.624 ^b^	0.669 ^b^	0.339 ^a^
C16 PALMITIC **	18.356 ^a^	20.161 ^b^	18.472 ^a^
C17 MARGARIC ***	0.918 ^b^	2.003 ^c^	0.328 ^a^
C18 ***	5.322 ^b^	5.855 ^c^	4.547 ^a^
C19 *	0.143 ^b^	0.246 ^c^	0.058 ^a^
C20 **	0.517 ^b^	0.572 ^b^	0.239 ^a^
C21	0.020 ^a^	0.063 ^a^	0.045 ^a^
C22 **	0.024 ^a^	0.110 ^b^	0.222 ^c^
C24 LIGNOCERIC ***	0.000 ^a^	0.015 ^a^	4.107 ^b^
SFA ***	29.155 ^a^	32.171 ^c^	31.297 ^b^
C14:1	0.037 ^a^	0.015 ^a^	0.043 ^a^
C15:1	0.060 ^b^	0.028 ^ab^	0.005 ^a^
C16:1 ***	3.025 ^a^	3.697 ^b^	3.743 ^c^
C17:1 **	0.395 ^b^	0.590 ^c^	0.230 ^a^
C20:1 ***	4.057 ^c^	0.174 ^a^	2.211 ^b^
C24:1 ***	0.700 ^b^	1.295 ^c^	0.457 ^a^
C18:1 n-9 OLEIC ***	16.581 ^b^	21.863 ^c^	2.563 ^a^
tC18:1 n-9 ***	0.387 ^b^	0.044 ^a^	0.724 ^c^
C18:1 n-11 VACCENIC ***	2.257 ^b^	0.015 ^a^	24.735 ^c^
C22:1 n-9 ***	0.488 ^b^	0.628 ^c^	0.027 ^a^
MUFA ***	27.601 ^a^	28.572 ^b^	34.932 ^c^
C18:2 n-6 LINOLEIC ***	4.111 ^b^	1.409 ^a^	11.981 ^c^
tC18:2 n-6 **	0.078 ^a^	0.034 ^a^	0.244 ^b^
C18:3 n-3 ALA ***	0.141 ^a^	3.302 ^c^	2.961 ^b^
C18:3 n-6 *	0.091 ^a^	0.071 ^a^	0.176 ^b^
C20:2 n-3 *	0.155 ^ab^	0.219 ^b^	0.106 ^a^
C20:2 n-6 **	0.439 ^a^	0.390 ^a^	0.739 ^b^
C20:3 n-3 ***	0.069 ^a^	1.100 ^c^	1.037 ^b^
C20:3 n-6 ***	2.436 ^c^	1.399 ^a^	1.438 ^b^
C20:4 n-6 ***	1.205 ^b^	0.062 ^a^	0.127 ^a^
C20:5 n-3 EPA ***	6.719 ^b^	6.969 ^c^	4.107 ^a^
C22:2 n-6 ***	0.667 ^b^	0.911 ^c^	0.145 ^a^
C22:5 n-3 ***	1.260 ^a^	1.698 ^c^	1.416 ^b^
C22:6 n-3 DHA ***	24.019 ^c^	21.694 ^b^	9.295 ^a^
PUFA ***	41.312 ^c^	39.257 ^b^	33.771 ^a^
∑Ω3 ***	32.363 ^b^	34.982 ^c^	18.922 ^a^
∑Ω6 ***	9.028 ^b^	4.275 ^a^	14.849 ^c^
∑Ω6/∑Ω3 ***	0.279 ^b^	0.122 ^a^	0.785 ^c^
P/S RATIO ***	1.417 ^c^	1.220 ^b^	1.079 ^a^

Different letters show significant differences among columns. * *p* < 0.05; ** *p* < 0.01; *** *p* < 0.001.

**Table 3:** Physicochemical results of the developed experimental products in contrast with control biscuits.

	CONTROL	FM-2.5	FM-5	FPH-2.5	FPH-5
Fracturability (g)	1.50	1.45	1.28	1.08	0.95 **
Hardness (g)	2318.04	1835.40	2053.66	2231.10	1835.58
L*	68.76	65.72	63.34 *	56.84 ***	55.44 ***
a*	2.74	4.84	6.27 *	9.69 ***	11.41 ***
b*	23.89	26.70	28.29 *	31.63 ***	32.15 ***
∆E	-	5.02 **	7.86 **	14.88 **	19.23 **
Acidity Index (Oleic acid %)	0.07	0.09 **	0.12 **	0.10	0.14
TBARS (mg/kg MDA)	0.09	0.19 **	0.28 **	0.24	0.27
Moisture %	4.60	4.69	4.69	5.31	5.34
a_w_	0.4543	0.4363	0.4363	0.4493	0.4493

FPH: fish protein hydrolysate; FM: fishmeal. The numbers 2.5 and 5 refer to % wheat flour substitution. * *p* < 0.05; ** *p* < 0.01; *** *p* < 0.001.

## Data Availability

All data generated or analyzed during this study are included in this published article.
